# Frequencies of Inaudible High-Frequency Sounds Differentially Affect Brain Activity: Positive and Negative Hypersonic Effects

**DOI:** 10.1371/journal.pone.0095464

**Published:** 2014-04-30

**Authors:** Ariko Fukushima, Reiko Yagi, Norie Kawai, Manabu Honda, Emi Nishina, Tsutomu Oohashi

**Affiliations:** 1 Department of Liberal Arts, The Open University of Japan, Chiba, Japan; 2 Department of Early Childhood Education, Tokyo Seitoku College, Tokyo, Japan; 3 Department of Research and Development, Foundation for Advancement of International Science, Tsukuba, Japan; 4 Research Council, Waseda University, Tokyo, Japan; 5 Department of Functional Brain Research, National Center of Neurology and Psychiatry, Kodaira, Japan; 6 Department of Cyber Society and Culture, School of Cultural and Social Studies, The Graduate University for Advanced Studies (SOKENDAI), Kanagawa, Japan; UNLV, United States of America

## Abstract

The hypersonic effect is a phenomenon in which sounds containing significant quantities of non-stationary high-frequency components (HFCs) above the human audible range (max. 20 kHz) activate the midbrain and diencephalon and evoke various physiological, psychological and behavioral responses. Yet important issues remain unverified, especially the relationship existing between the frequency of HFCs and the emergence of the hypersonic effect.

In this study, to investigate the relationship between the hypersonic effect and HFC frequencies, we divided an HFC (above 16 kHz) of recorded gamelan music into 12 band components and applied them to subjects along with an audible component (below 16 kHz) to observe changes in the alpha2 frequency component (10–13 Hz) of spontaneous EEGs measured from centro-parieto-occipital regions (Alpha-2 EEG), which we previously reported as an index of the hypersonic effect.

Our results showed reciprocal directional changes in Alpha-2 EEGs depending on the frequency of the HFCs presented with audible low-frequency component (LFC). When an HFC above approximately 32 kHz was applied, Alpha-2 EEG increased significantly compared to when only audible sound was applied (positive hypersonic effect), while, when an HFC below approximately 32 kHz was applied, the Alpha-2 EEG decreased (negative hypersonic effect). These findings suggest that the emergence of the hypersonic effect depends on the frequencies of inaudible HFC.

## Introduction

It is generally accepted that humans cannot perceive air vibrations in the frequency range above 20 kHz as sound. Oohashi et al. reported, however, that a non-stationary sound containing significant quantities of high-frequency components (HFC) beyond the human audible range evokes a significant increase in the regional cerebral blood flow (rCBF) in the midbrain and the thalamus [1], [2] and in the occipital alpha frequency component of spontaneous electroencephalogram (EEG) as compared with an otherwise identical sound from which the HFCs are removed [1], [3], [4]. In addition, the inclusion of HFCs renders a sound more pleasant [1]–[3] and evokes a specific behavior, that is, the listener spontaneously increases the comfortable listening level (CLL) of the presented sound [2]–[4]. We call such phenomena collectively “the hypersonic effect.” The phenomena induced by the inclusion of HFCs in EEG [5] and the resulting subjective impression [6] have been replicated by other research groups. The hypersonic effect is induced only when HFCs are presented to the listener's entire body surface but not when presented exclusively to the listener's ear [7].

The discovery of this effect, which was reported for the first time at the 91st Audio Engineering Society convention in 1991 by Oohashi et al., has greatly impacted the audio industry; cutting edge digital audio media, such as the super audio compact disc (SACD), digital versatile disc audio (DVD-Audio), and Blu-ray Disc, allow the recording of inaudible HFCs. Additionally, internet-based, high-resolution audio distribution has now taken off, attracting international interest in the effect of inaudible HFCs on sound quality. However, the rampant variety of audio formats with no agreement as to the requisite frequency bands responsible for sound quality have resulted in inconsistent specifications for a recordable and reproducible frequency range among the diverse assortment of digital media. This situation is partly caused by the fact that frequency of HFCs necessary for the emergence of the hypersonic effect has yet to be systematically examined.

It is well known that HFCs with specific structure ranging between audible and inaudible frequencies induce avoidance behaviors in humans (e.g., the so-called mosquito alarm [8], [9]). Contrary to the hypersonic effect, the mosquito alarm may arouse a sense of displeasure in humans. Therefore it is incumbent on life science research to delve into how differences in the frequency of applied sounds affects the physiology of humans.

In the present study, we applied segments of an HFC divided at arbitrary frequencies and observed the alpha2 component (10–13 Hz) of spontaneous EEG recorded from the centro-parieto-occipital regions (Alpha-2 EEG), which is recognized as an index of the emergence of the hypersonic effect [1], [3], [7], [10]. Accordingly, we examined the influence of different HFCs frequencies on Alpha-2 EEG.

## Methods

### Subjects

In Experiment 1, 12 healthy Japanese volunteers (6 males and 6 females; ages 20–71) participated. In Experiment 2, as explained later, 12 sub-experiments were carried out on different days. Ten subjects from a subject pool of 19 healthy Japanese volunteers (9 males and 10 females; ages 20–71) took part in each sub-experiment. Each of the 19 subjects participated in 6.3±3.4 (SD) of these sub-experiments on average.

After each experiment, the subjects completed a questionnaire on their sleep and overall physical condition, as follows:

*My feeling during the experiment (subject excluded if answered “not so good”)*

*My physical condition today (subject excluded if answered “not good” or “bad”)*

*How much sleep I got last night (subject excluded if answered “less than 4 hours”)*

*How many hours before the experiment I woke up (subject excluded if answered “less than 2 hours” or “more than 8 hours”)*

Subjects were excluded that day if they answered any one of these 4 questions in the manner indicated.

The data from 11 out of 12 subjects were analyzed for Experiment 1. The data from 7 to 10 out of 10 subjects were analyzed for each sub-experiment of Experiment 2. The number of valid subjects for each sub-experiment is given under Results. None of the subjects had any history of neurological or psychiatric disorder. Written informed consent was obtained from all subjects prior to the study. Experiments were carried out with the approval of the ethics committee at the National Center of Neurology and Psychiatry.

### Sound material

We obtained a 200-second experimental audio sample of gamelan orchestral music on Bali Island, Indonesia, having drawn on our preliminary studies there on the hypersonic effect. The gamelan music was newly recorded live on location using an originally developed system, with a high-speed, one-bit coding signal processor [11] in Direct Stream Digital (DSD) format having a sampling frequency of 5.6448 MHz. The electrical signal contained a wealth of HFCs, even reaching 100 kHz and above.

### Sound presentation system

**Figure 1:**
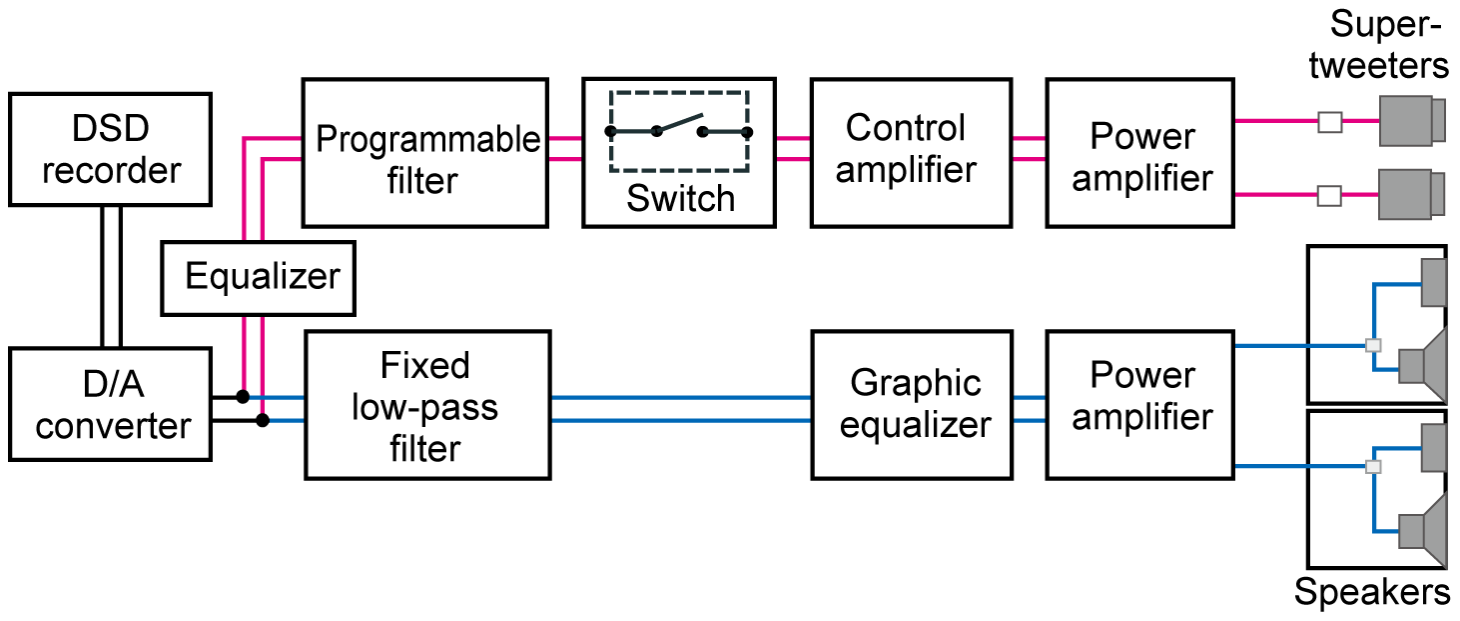
Block diagram of the frequency-variable sound presentation system. Each signal of the stereophonic sound material was divided into a low frequency component (LFC) and a high frequency component (HFC). The LFC was low-pass filtered at the cut off frequency of 16 kHz, while the HFC was band-pass filtered at arbitrary frequencies over 16 kHz by a programmable band-pass filter. The LFC and the HFC were independently amplified and reproduced through speakers and super-tweeters. Either LFC alone (control) or the LFC and an HFC together was applied to the subjects to observe the emerging state of the hypersonic effect.

To apply the sound material, we constructed a frequency-variable sound presentation system (Figure 1), based on our bi-channel sound presentation system [1] in which audible low-frequency components (LFCs) and inaudible HFCs of the experimental sound source were divided by frequency, then independently amplified and transduced into air vibrations. In the present study, the LFCs were extracted with a low-pass filter (CF-6FL, NF Electronic Instruments, Yokohama, Japan) having a cutoff frequency of 16 kHz and a cutoff attenuation of 80 dB/octave. The HFCs were extracted with programmable filters (FV-661 and 3628, NF Electronic Instruments, Yokohama, Japan) having cutoff attenuations of 90 dB/octave and 70 dB/octave respectively, to low-pass, high-pass or band-pass the sound source at any arbitrary cutoff frequencies. The pass-band ripples of all filters were ±1dB. The LFCs were transduced into air vibration by an OOHASHI MONITOR Op.1 (designed by author TO). The HFCs were presented by super-tweeters (PT-R9, Pioneer Co., Ltd., Kanagawa, Japan, and printed-ribbon tweeters originally developed by us).

### Experimental design

We carried out two experiments in which we applied different kinds of HFCs.

#### Experiment 1. Broad examination of the frequency dependency of the hypersonic effect

**Figure 2:**
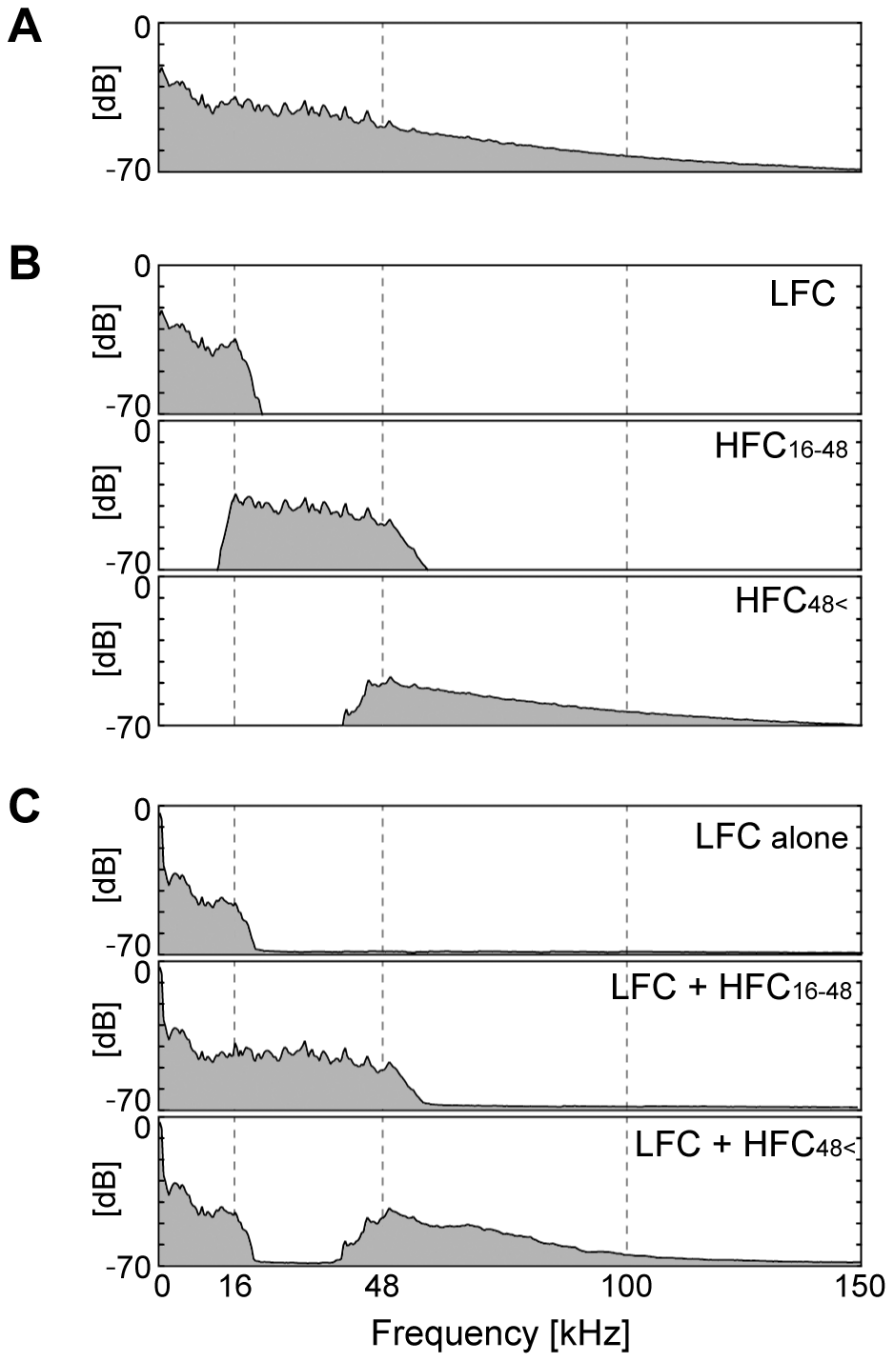
Averaged power spectra of sound materials applied in Experiment 1. A: Electric signal of the sound source. B: Filtered electric signals of LFC under 16 kHz (control), HFC at 16–48 kHz, and HFC at 48 kHz <. C: The sounds and air vibration reproduced by the frequency-variable sound application system. Power was calculated from the data recorded at the subject's position. Faithful reproductions from electric signals to air vibrations were observed.

In Experiment 1 we broadly tested whether the difference of frequencies of the applied HFCs affected the emergence of the hypersonic effect. We divided the HFC into two components at 48 kHz, creating HFC_16-48_ and HFC_48 <_. HFC subscripts indicate the frequency (kHz) of each bandwidth. The reason for dividing the HFCs in this way was to examine if the widely-used sampling frequency of high resolution digital audio, 96 kHz, which can record and reproduce sounds up to 48 kHz, effectively induced hypersonic effects in the subjects. We then applied each of the two HFCs together with a LFC under 16 kHz. We also applied the same LFC by itself, to serve as a control. The power spectra of the electrical signal of each component as well as those of the presented sounds are shown in Figure 2. We then compared three conditions: [LFC+HFC_16-48_], [LFC+HFC_48<_] and the control (LFC alone with no HFC). These three application conditions were presented in an ABCCBA sequence, where each of these three sound conditions was assigned to A, B or C in a counterbalanced deployment across subjects. Subjects were exposed to the six sound presentations successively without interval except for a several-minute interval between the former and latter three presentations. We applied the 200-sec sound material to the subjects under each condition and recorded their EEGs.

#### Experiment 2. Detailed examination of the frequency dependency of the hypersonic effect

In Experiment 2 we applied narrower bandwidth HFCs to delve deeper into the frequency dependency of the hypersonic effect as observed in Experiment 1.

**Figure 3:**
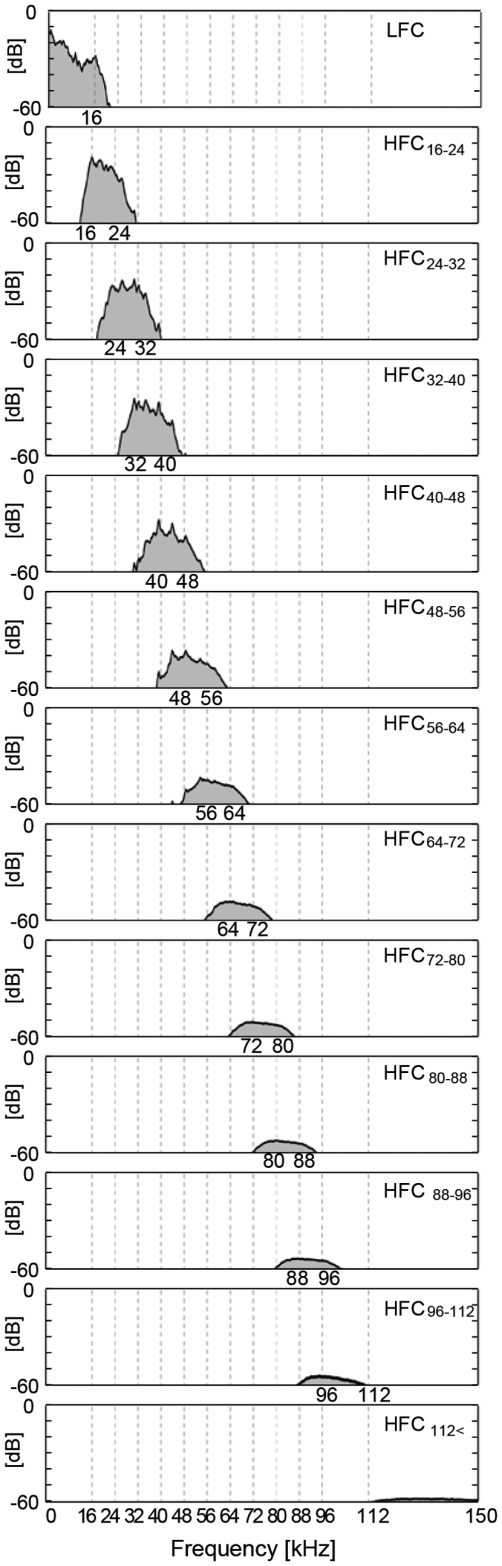
Averaged power spectra of sound materials applied in Experiment 2. Filtered electric signals of LFC under 16(control) and HFCs above 16 kHz. Frequency from 16 kHz to 96 kHz was divided into ten bandwidth components at every 8 kHz, while higher frequencies above 96 kHz were divided into two bandwidths, one at 96 kHz–112 kHz, the other at 112 kHz <. A filtered HFC was applied together with an LFC to the subjects before or after LFC alone (control) was applied.

The HFCs were divided into ten components at every 8 kHz bandwidth, starting from 16 kHz: HFC_16–24_, HFC_24–32_, HFC_32–40_, HFC_40–48_, HFC_48–56_, HFC_56–64_, HFC_64–72_, HFC_72–80_, HFC_80–88_, HFC_88–96_. Higher frequency HFCs above 96 kHz was subdivided into two bandwidths: HFC_96–112_, HFC_112<_. Thus there were twelve kinds of HFCs in all. The power spectra of the LFC and the HFCs are shown in Figure 3. Experiment 2 consisted of 12 sub-experiments, each corresponding to one of the above-cited 12 kinds of HFCs. These experiments were performed on different days to avoid subject fatigue. In each experiment, an HFC with LFC (for example, [LFC+HFC_16–24_]) and the control ([LFC] alone) were presented in pairs four times. The conditions were presented in an ABBABAAB sequence. [LFC+HFC] and [LFC] were assigned as A and B, or B and A, respectively, in counterbalanced deployment across subjects. Subjects were exposed to the eight sound presentations without interval except for a several-minute interval between the former and latter four trials.

### EEG measurement and analysis

EEG recordings and analysis were carried out in conformity with our previous studies [1], [3], [7]: a telemetric system was employed for the EEG recording to minimize subject restraint. These data were recorded from 12 scalp electrodes (Fp1, Fp2, F7, Fz, F8, C3, C4, T5, Pz, T6, O1 and O2) according to the International 10–20 System using linked earlobe electrodes as a reference. Two-sec analysis epochs were set at each 1 sec with a 1-sec overlap to calculate FFT with a sampling frequency of 256 Hz and 0.5 Hz frequency resolution to obtain the power spectra of each electrode. If a 2-sec analysis epoch contained an artifact, we eliminated it from successive analysis. The square root of the averaged power level in the frequency range of 10.0–13.0 Hz at each electrode was set as the equivalent potential of the EEGs in the alpha2 frequency band. To eliminate inter-subject variability, the potentials acquired from each electrode of a subject were normalized with respect to the mean value across all time epochs, electrodes, and experimental conditions in the subject. The potentials obtained from the 7 electrodes in the centro-parieto-occipital region (C3, C4, T5, Pz, T6, O1 and O2) were averaged across all analysis epochs for each condition and of each subject. In the previous studies, it is reported that, when the hypersonic effect emerged, changes in EEG appeared only dozens of secs after the onset of the sound application and remained for nearly 100 secs after the end of the application [1]. Therefore, as in our previous series of studies [1], [7], the above value obtained from EEGs during the latter 100 secs of a 200-sec sound presentation was labeled, for further analysis, as Alpha-2 EEG.

In Experiment 1 we performed univariate analysis of variance (ANOVA) using Alpha-2 EEG as a variable and the HFC condition as a factor followed by a Tukey's post-hoc test.

For all sub-experiments in Experiment 2, ΔAlpha-2 EEG was calculated by subtracting the Alpha-2 EEG of the control from that of [LFC+HFC]. Univariate ANOVA was performed using ΔAlpha-2 EEG as a variable and the frequency of the HFC as a factor followed by a Tukey's post-hoc test. Then, as precisely explained in Results, the data of the sub-experiments were divided into two groups, those with HFCs below 32 kHz and those with HFCs above 32 kHz, based on the observation that the mean of ΔAlpha-2 EEG gradually changed from negative to positive as the frequency of the HFCs increased, crossing the zero-line at around 32 kHz. We performed statistical tests for the two groups: unpaired t-test to compare between the two groups and 1-sampled t-test within each group to examine if the average ΔAlpha-2 EEG of each group significantly differed from zero.

## Results

### Experiment 1. Broad examination of the frequency dependency of the hypersonic effect

Of the 12 subjects who participated in Experiment 1, one whose physical condition “was not good” on the day was eliminated and 11 were considered valid (6 males and 5 females; mean age of 44.5±14.1). Among the 100 analysis epochs, 98.5±1.9 (mean±SD) artifact-free epochs were valid.

**Figure 4:**
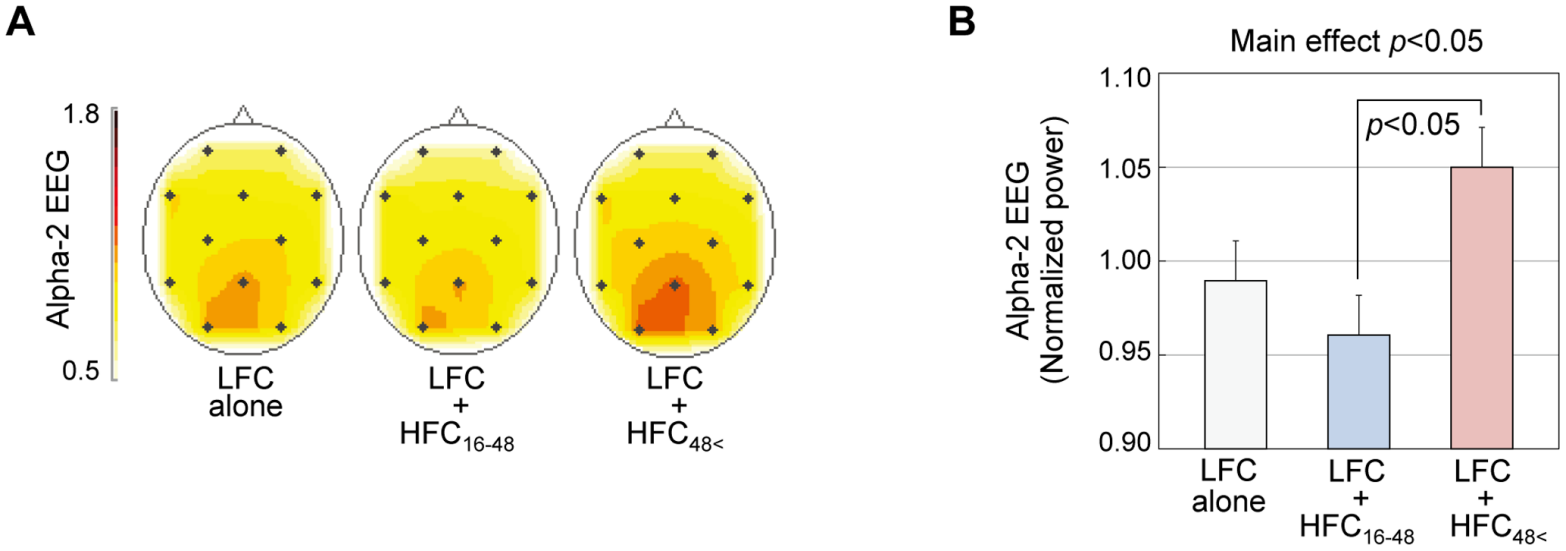
Results of Experiment 1: broad examination of frequency dependency of the hypersonic effect. A: Scalp distribution of electroencephalographic activity during application of different HFC frequencies. To overview the spatial distribution of the equivalent potential of alpha2 frequency band (10–13 Hz) of EEGs, colored contour line maps were constructed by using 2,565 scalp grid points computed by linear interpolation and extrapolation of alpha2 components from 12 electrodes [33], [34]. Darker red indicates higher alpha2. Note that the alpha2 in the occipital region changed depending on the frequency of the HFC. B: Mean (+SE) value of Alpha-2 EEGs. Potential of alpha2 frequency band recorded from 7 electrodes in the centro-parieto-occipital region (C3, C4, T5, Pz, T6, O1 and O2) for the last 100 sec of sound application was averaged across all subjects. Analysis of variance (ANOVA) revealed the main effect of HFC to be significant, and Tukey's post-hoc test found significant difference between [LFC+HFC_16-48_] and [LFC+HFC_48<_].

Figure 4A shows the scalp distribution of the equivalent potentials of alpha2 frequency band averaged over all subjects for each of the three conditions. The alpha2 EEGs were observed to be dominant in the occipital area throughout the experiments. Figure 4B shows the mean and SE of Alpha-2 EEG of the subjects, recorded for each condition. When [LFC+HFC_48<_] was applied, Alpha-2 EEG increased compared to when the control was applied. On the contrary, when [LFC+HFC_16–48_] was applied, Alpha-2 EEG decreased. We performed ANOVA, which showed significant main effect of HFC condition (*F*(2,30) = 4.261, *p*<0.05). The Tukey's post-hoc test revealed that the Alpha-2 EEGs differed with statistical significance between the application of [LFC+HFC_16–48_] and [LFC+HFC_48<_] (*p*<0.05), although neither [LFC+HFC_16–48_] nor [LFC+HFC_48<_] showed significant difference in comparison with the control. Alpha-2 EEG decreased in 6 of 11 subjects under [LFC+HFC_16–48_], compared with the control, while it increased in 7 of 11 subjects under [LFC+HFC_48<_], compared with the control.

### Experiment 2. Detailed examination of the frequency dependency of the hypersonic effect

The number, sex and age of valid subjects for each of the applied HFCs were as follows: HFC_16–24_: M5, F5, age 20–71; HFC_24–32_: M7, F2, age 20–54; HFC_32–40_: M4, F3, age 20–52; HFC_40–48_: M6, F3, age 20–52; HFC_48–56_: M3, F4, age 20–71; HFC_56–64_: M6, F3, age 39–71; HFC_64–72_: M6, F3, age 20–71; HFC_72–80_: M5, F2, age 33–71; HFC_80–88_: M6, F2, age 20–71; HFC_88–96_: M8, F2, age 33–71; HFC_96–112_: M7, F3, age 33–71; HFC_112<_: M5, F4, age 33-52. Out of all 100-sec analysis epochs, 95.8±7.2 (mean±SD) artifact-free epochs were valid. Mean of artifact-free epochs were within 90.7 to 99.2 sec for all sub-experiments.

**Figure 5:**
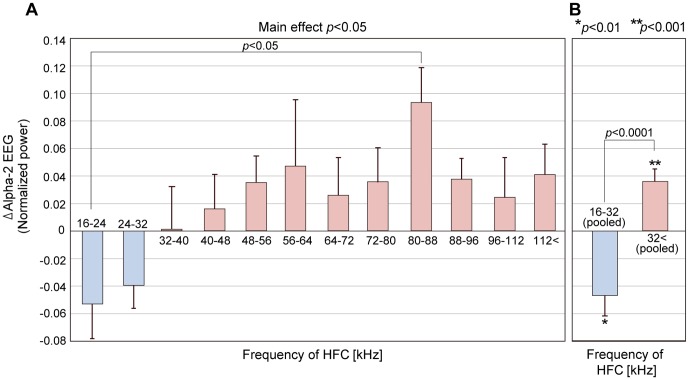
Results of Experiment 2: detailed examinations of frequency dependency of the hypersonic effect. A: Mean (+SE) values of ΔAlpha-2 EEG across the subjects in each of twelve sub-experiments. The frequencies indicate the frequency range of HFC that were applied together with LFC ([LFC+HFC]) in comparison with the control ([LFC] alone). ΔAlpha-2 EEG is calculated by subtracting Alpha-2 EEG obtained during [LFC] from those during [LFC+HFC] in each subject. Univariate ANOVA showed the main effect of the frequency of HFC (*p*<0.05). Tukey's post-hoc tests showed a significant difference between ΔAlpha-2 EEG obtained with [LFC+HFC_16-24_] and that obtained with [LFC+HFC_80–88_] (*p*<0.05). B: Comparison of ΔAlpha-2 EEG between two groups of pooled data obtained in the sub-experiments using HFC below 32 kHz and those obtained in the sub-experiments using HFC above 32 kHz. Unpaired t-tests showed significant difference between the two groups and 1-sampled t-tests showed significant difference from zero for each group, that is, ΔAlpha-2 EEG obtained by using HFC below 32 kHz was significantly negative (*p*<0.01), while those obtained by using HFC above 32 kHz was significantly positive (*p*<0.001).

Figure 5A shows the ΔAlpha-2 EEGs averaged over subjects and the SE in each sub-experiment, arranged in order of frequency of the applied HFC. The positive ΔAlpha-2 EEG means that Alpha-2 EEG increased in comparison to the control by inclusion of the HFC, while the negative ΔAlpha-2 EEG means the decreased Alpha-2 EEG by inclusion of the HFC. Accordingly, Figure 5A shows that Alpha-2 EEG reversed direction according to the frequency of the HFCs. When the relatively lower HFCs of 16–24 kHz and 24−32 kHz were applied, Alpha-2 EEG decreased (that is, ΔAlpha-2 EEG was negative); when the HFC reached the boundary at around 32 kHz, it became neutral; when higher HFCs above 40 kHz were presented, Alpha-2 EEG increased for [LFC+HFC] compared with the control (that is, ΔAlpha-2 EEG was positive).

We carried out ANOVA using ΔAlpha-2 EEG as a variable and the frequency of the HFC as a factor. Results showed the main effect of the frequency of the applied HFC was significant (*F*(11,92) = 1.980, *p*<0.05). The Tukey's post-hoc test showed that ΔAlpha-2 EEG differed with statistical significance between the application of [LFC+HFC_16–24_], which marked the lowest mean, and [LFC+HFC_80–88_], the highest (*p*<0.05). The application of [LFC+HFC_24–32_], the second lowest, and that of [LFC+HFC_80–88_] did not show significant difference but the *p*-value marked close to the significance level (*p* = 0.06). In the experiment using HFC_80–88_, 8 out of 9 subjects showed positive ΔAlpha-2 EEG. On the contrary, 8 out of 10 subjects in the experiment using HFC_16–24_ and 8 out of 9 subjects in the sub-experiment using HFC_24–32_ showed negative ΔAlpha-2 EEG.

Having observed that the mean value of ΔAlpha-2 EEG became higher as the frequency of the applied HFC increased and changed from negative to positive at around 32 kHz, we pooled and divided the data into two groups, one for HFC below 32 kHz (n = 19) and the other for HFC above 32 kHz (n = 85) (Figure 5B). Unpaired t-tests (two-sided test) showed that there was significant difference between the two groups (*t*(103) = 4.06, *p*<0.0001). We also performed 1-sampled t-tests (two-sided test) for each group. The ΔAlpha-2 EEG below 32 kHz was significantly negative (*t*(18) = −3.082, *p*<0.01), while that above 32 kHz was significantly positive (*t*(84) = 4.014, *p*<0.001).

## Discussion

Our two experiments suggest that the appearance of the hypersonic effect markedly changes depending on the frequency of the HFCs when applied along with lower audible components. Experiment 2, in particular, revealed that Alpha-2 EEG, used as an index of the emergence of the hypersonic effect, indicates contrary behaviors between higher and lower frequencies of applied HFC, at around 32 kHz; it decreases with lower frequency HFCs and increases with higher frequency HFCs. Furthermore, the magnitude of Alpha-2 EEG gradually changes according to the frequency of the HFCs. This study is the first to show that the emergence of the hypersonic effect depends on the frequency of the HFCs and that its effect may be either positive or negative.

In Experiment 1, we broadly examined the frequency dependency of the hypersonic effect. We found significant difference in Alpha-2 EEG between LFC associated with HFC below 48 kHz (i.e., LFC+HFC_16–48_) and that above 48 kHz (i.e., LFC+HFC_48<_). However, the difference was not significant when Alpha 2-EEG in LFC+HFC_16–48_ and LFC+HFC_48<_ were compared to the control, LFC alone, although there was a tendency for Alpha2-EEG to decrease in HFC_16–48_ while it increased in HFC_48<_. One possible reason could be that HFC_16–48_ includes contrary components, one decreasing and the other increasing Alpha 2-EEG and thereby cancelling each other out.

In these experiments, the total power of sound was always larger in LFC+HFC than in LFC alone. Thus, it is possible to assume that changes in Alpha-2 EEG are related to the total power of applied sound rather than to frequency. In fact, it has been reported that increasing the intensity of inaudible HFCs enhances the hypersonic effect with a significant increase in the occipital alpha EEG and in the CLL of subjects [3], [4]. To examine that possibility, we plotted power of sound stimuli and ΔAlpha-2 EEG against frequency in Figure S1. The total power was largest when HFCs of 24–32 kHz, which reduced ΔAlpha-2 EEG, were applied. On the other hand, it was much smaller when HFCs of 80–88 kHz, which showed the greatest increase of ΔAlpha-2 EEG, were applied. Thus it is difficult to explain the behavior of ΔAlpha-2 EEG in terms of the total power of sound.

Based on these findings, we have called the decrease in Alpha-2 EEG and other related phenomena observed when HFCs of 16–32 kHz are applied *the negative hypersonic effect*, to distinguish it from what we previously called the hypersonic effect, and what now we call *the positive hypersonic effect*, as appropriate.

Other studies have indicated a positive correlation between the occipital alpha EEG component and the rCBF of deep-lying brain regions [1], [12]–[17], including the midbrain and the thalamus, which are reportedly activated in the hypersonic effect [1]. We also reported that the rCBF of these regions showed strong correlation with the occipital alpha2 EEG component, which is the faster component of the alpha rhythm (10–13 Hz) [10]. Since many factors contribute to the generation of alpha oscillations (e.g., [18]), we cannot determine a causal relationship between the changes in the Alpha-2 EEG observed in this study and the neuronal activity of the deep-lying brain structure. Omata et al. [17] reported, based on a simultaneous recording of EEG and fMRI, that the slow fluctuation component of alpha power time series with frequencies slower than 0.04 Hz was positively correlated with activity in the midbrain, the medial part of the thalamus and anterior cingulate cortex, which work as part of the reward-generating neuronal network, while the fast fluctuation component, such as waxing and waning [19], correlated with the lateral part of the thalamus. In this study, since we averaged Alpha-2 EEG over 100-sec analysis epoch, the observed changes are considered to correspond to the slow fluctuation component as examined in the Omata study. Of course, we do not argue that changes in EEG observed in this study uniquely reflect the activity of the reward-generating system since such structures likewise contribute to various cognitive functions other than reward generation. Nevertheless, considering the fact that the hypersonic effect involves the enhancement of the pleasure sensation of sound [1]–[3] and induces an approaching behavior [2]–[4], we envisage the possibility that the changes in EEG observed in this study may pertain to a certain relationship with the activity of the reward-generating neuronal network.

The mechanism explaining how a difference in the frequency of HFCs induces contrary behaviors of Alpha-2 EEG is not yet known, but we may look to rodent vocalization for reference, especially with regard to the relationship between the frequency of ultrasonic vocalization of rats and their affective reactions. The 22 kHz vocalization of rats relates negative affectation and activation of the punishment-relating neuronal network [20]–[23], inducing avoidance behavior [20], [23]–[27], while the 50 kHz vocalization relates positive affectation and activation of the reward-generating neuronal network [21]–[23], [28]–[30], inducing approach behavior [23], [31]. As is widely known, the audible frequency of rats reaches a much higher frequency than that of humans; all of the frequencies used in these studies are within the audible range of rats. We cannot, therefore, conclude any common mechanism between rodent vocalization and what was observed in this study. However, these studies may provide a clue for subsequent investigation.

As for the positive hypersonic effect, HFCs at around 80−88 kHz induce the maximum activity of Alpha-2 EEG. Such frequencies are within the ultra-high frequency domain, which is far beyond and not contiguous to the 20 kHz upper limit of the human audible range. The authors had not anticipated that human brain activity would sharply respond to such ultra-high HFCs. Furthermore, the application of even higher HFCs, such as 96–112 kHz or even over 112 kHz, which are extremely faint in power, also increased Alpha-2 EEG no less than did HFCs of 40–48 kHz and 48–56 kHz. Such data imply the existence of unknown human sensitivity to high frequency air vibrations, which may further contribute to discussion in the basic neuroscience field in light of the discovery of the hypersonic effect [32].

Such widely ranging sensitivity of humans to high frequency components causing a hypersonic effect calls for a re-examination of current audio formats such as CDs, SACDs, DVD-Audios, even of audio formats with higher resolution. The digital audio formats with a reproducible upper frequency below 32 kHz cannot induce a positive hypersonic effect and may not contribute to improved sound quality. If a negative hypersonic effect, observed when HFCs between 16 kHz and 32 kHz were applied, may pertain to a certain relationship with a decrease in the activity of the deep-lying brain structure, a biological assessment would be required in terms of the safe use of HFCs. Needless to say, not only audio formats but also the content itself should be examined to see if it contains a sufficient amount of higher HFCs. To utilize digital audio technologies effectively and safely, it is thus of utmost urgency to re-examine current audio formats in terms of the latest advances in life science.

## Conclusion

By observing Alpha-2 EEG, it became clear that the emergence of the hypersonic effect changes either positively or negatively depending on the frequency of the HFC applied along with the audible sound. We showed that Alpha-2 EEG increases when HFCs above approximately 32 kHz are applied, which indicates that a positive hypersonic effect has emerged, as shown in our earlier studies. Our present study reports, for the first time, that Alpha-2 EEG decreases when HFCs below approximately 32 kHz are applied, which indicates the emergence of a negative hypersonic effect.

## Supporting Information

Figure S1
**The powers of the applied sound stimuli and the change of Alpha-2 EEG.** To examine whether the change of Alpha-2 EEG was dependent on the total power of sound stimuli, the power of each frequency component of each sound stimulus was calculated as partial over all (POA) and plotted with ΔAlpha-2 EEG in Experiment 2. Each bar represents POA of LFC and HFC in each sound stimulus. POA of LFC (gray) is constant across all sub-experiments since identical LFC was always used, while POA of HFC (green) varies across sub-experiments. ΔAlpha-2 EEG was plotted in a red line. POA is a power between specific frequencies and calculated as follows. 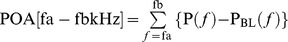 Here, fa and fb is the lower and upper limit of the frequency range to be analyzed, respectively. P(*f*) and P_BL_(*f*) is the averaged power spectrum for 200 sec of the gamelan music used in the present study and that of the background noise at frequency *f*, respectively. P(*f*) and P_BL_(*f*) were calculated from 0 to 150 kHz using FFT analyzer. Since a slope of power spectrum existed outside the analysis frequency range according to the filter characteristics, the power of such components were also included. Therefore, POA corresponds to an area of power spectrum between specific frequencies.(TIF)
