# Supplementary figures and images for: Frequencies of Inaudible High-Frequency Sounds Differentially Affect Brain Activity: Positive and Negative Hypersonic Effects
Source: PLoS One. 2014 Apr 30;9(4):e95464. doi: 10.1371/journal.pone.0095464 (PMC4005747; doi:10.1371/journal.pone.0095464)

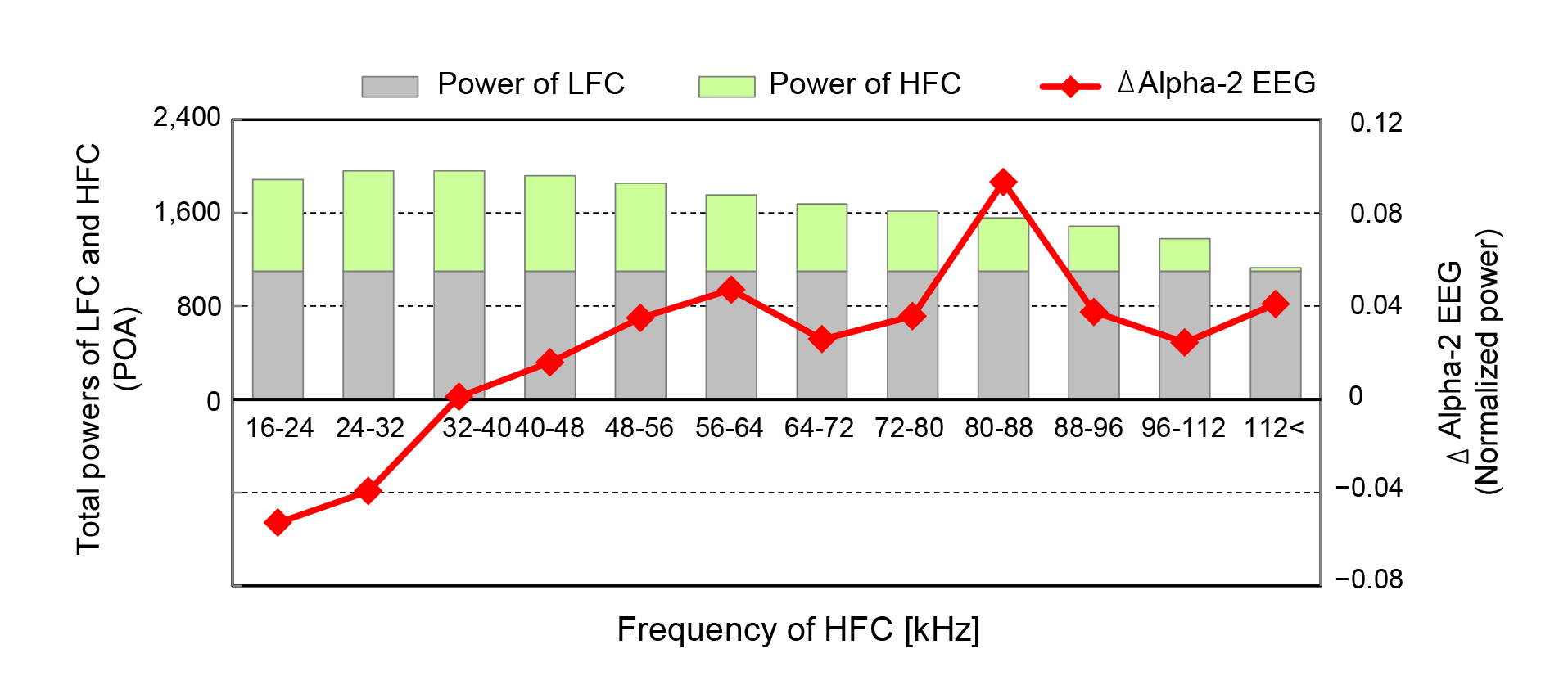

Supplement: Figure S1 — The powers of the applied sound stimuli and the change of Alpha-2 EEG. To examine whether the change of Alpha-2 EEG was dependent on the total power of sound stimuli, the power of each frequency component of each sound stimulus was calculated as partial over all (POA) and plotted with ΔAlpha-2 EEG in Experiment 2. Each bar represents POA of LFC and HFC in each sound stimulus. POA of LFC (gray) is constant across all sub-experiments since identical LFC was always used, while POA of HFC (green) varies across sub-experiments. ΔAlpha-2 EEG was plotted in a red line. POA is a power between specific frequencies and calculated as follows. Here, fa and fb is the lower and upper limit of the frequency range to be analyzed, respectively. P(f) and PBL(f) is the averaged power spectrum for 200 sec of the gamelan music used in the present study and that of the background noise at frequency f, respectively. P(f) and PBL(f) were calculated from 0 to 150 kHz using FFT analyzer. Since a slope of power spectrum existed outside the analysis frequency range according to the filter characteristics, the power of such components were also included. Therefore, POA corresponds to an area of power spectrum between specific frequencies. (TIF) [file pone.0095464.s001.tif]
